# Statistical analysis plan for early goal-directed therapy using a physiological holistic view - the ANDROMEDA-SHOCK: a randomized controlled trial

**DOI:** 10.5935/0103-507X.20180041

**Published:** 2018

**Authors:** Glenn Hernández, Alexandre Biasi Cavalcanti, Gustavo Ospina-Tascón, Arnaldo Dubin, Francisco Javier Hurtado, Lucas Petri Damiani, Gilberto Friedman, Ricardo Castro, Leyla Alegría, Maurizio Cecconi, Jean-Louis Teboul, Jan Bakker

**Affiliations:** 1 Departamento de Medicina Intensiva, Facultad de Medicina, Pontificia Universidad Católica de Chile - Santiago, Chile.; 2 Instituto de Pesquisa, HCor-Hospital do Coração - São Paulo (SP), Brasil.; 3 Departamento de Medicina Intensiva, Fundación Valle del Lili, Universidad ICESI - Cali, Colômbia.; 4 Serviço de Terapia Intensiva, Sanatorio Otamendi y Miroli - Ciudad Autónoma de Buenos Aires, Argentina.; 5 Centro de Terapia Intensiva, Hospital Español, Escuela de Medicina, Universidad de la República - Montevidéu, Uruguai.; 6 Departamento de Medicina Interna, Faculdade de Medicina, Universidade Federal do Rio Grande do Sul - Porto Alegre (RS), Brasil.; 7 St George's University Hospitals NHS Foundation Trust - Londres, Reino Unido.; 8 Service de Réanimation Médicale, Hôpitaux Universitaires Paris-Sud, Assistance Publique-Hôpitaux de Paris - Paris, França.; 9 Division of Pulmonary, Allergy, and Critical Care Medicine, Columbia University Medical Center - Nova Iorque, Estados Unidos.; 10 Erasmus MC University Medical Center, Department Intensive Care Adults - Rotterdam, CA, Holanda.; 11 Division of Pulmonary, and Critical Care Medicine, New York University - Langone - Nova Iorque, Estados Unidos.

**Keywords:** Peripheral perfusion, Resuscitation, Shock, septic, Statistical analysis, Bias

## Abstract

**Background:**

ANDROMEDA-SHOCK is an international, multicenter, randomized controlled trial comparing peripheral perfusion-targeted resuscitation to lactate-targeted resuscitation in patients with septic shock in order to test the hypothesis that resuscitation targeting peripheral perfusion will be associated with lower morbidity and mortality.

**Objective:**

To report the statistical analysis plan for the ANDROMEDA-SHOCK trial.

**Methods:**

We describe the trial design, primary and secondary objectives, patients, methods of randomization, interventions, outcomes, and sample size. We describe our planned statistical analysis for the primary, secondary and tertiary outcomes. We also describe the subgroup and sensitivity analyses. Finally, we provide details for presenting our results, including mock tables showing baseline characteristics, the evolution of hemodynamic and perfusion variables, and the effects of treatments on outcomes.

**Conclusion:**

According to the best trial practice, we report our statistical analysis plan and data management plan prior to locking the database and initiating the analyses. We anticipate that this procedure will prevent analysis bias and enhance the utility of the reported results.

## INTRODUCTION

Early recognition of tissue hypoperfusion and its reversal in septic shock are key factors to improving survival rates.^(1)^ Hyperlactatemia has traditionally been considered to be a hallmark of ongoing tissue hypoxia;^(2)^ therefore, normalization of lactate levels has been recommended as a resuscitation target.^(3)^ However, other non-hypoperfusion-related causes of hyperlactatemia might be present in an unknown number of patients, leading to the risk of over-resuscitation.^(4)^

Peripheral perfusion could be used as a potential alternative resuscitation goal.^(5-8)^ The excellent prognosis associated with capillary refill time (CRT) recovery, its rapid response time to fluid loading, its relative simplicity, its availability in resource-limited settings, and its capacity to change in parallel with the perfusion of physiologically relevant territories constitute strong reasons to evaluate the usefulness of CRT as a guide for resuscitation in septic shock patients.

ANDROMEDA-SHOCK is an international, multicenter, randomized controlled trial comparing peripheral perfusion-targeted resuscitation (PPTR) to lactate-targeted resuscitation (LTR) in patients with septic shock in order to test the hypothesis that resuscitation aimed at peripheral perfusion will be associated with lower morbidity and mortality.

This article outlines the statistical analysis plan (SAP) for ANDROMEDA-SHOCK with the aim of preventing statistical analysis bias arising from exploratory analyses after the study results are known. The SAP was developed following appropriate guidelines^(9)^ prior to locking the trial database and starting analyses.

Our primary objective is to determine whether PPTR is associated with a lower 28-day mortality rate than that of LTR in patients with septic shock.

Our secondary objectives are to determine if, in patients with septic shock, PPTR compared to LTR can decrease all-cause mortality within 90 days; increase mechanical ventilation-free days, renal replacement therapy-free days, and vasopressor-free days within 28 days; decrease organ dysfunction at 72 hours; and decrease intensive care unit (ICU) and hospital length of stay.

## METHODS

### Trial design

**Figure 1:**
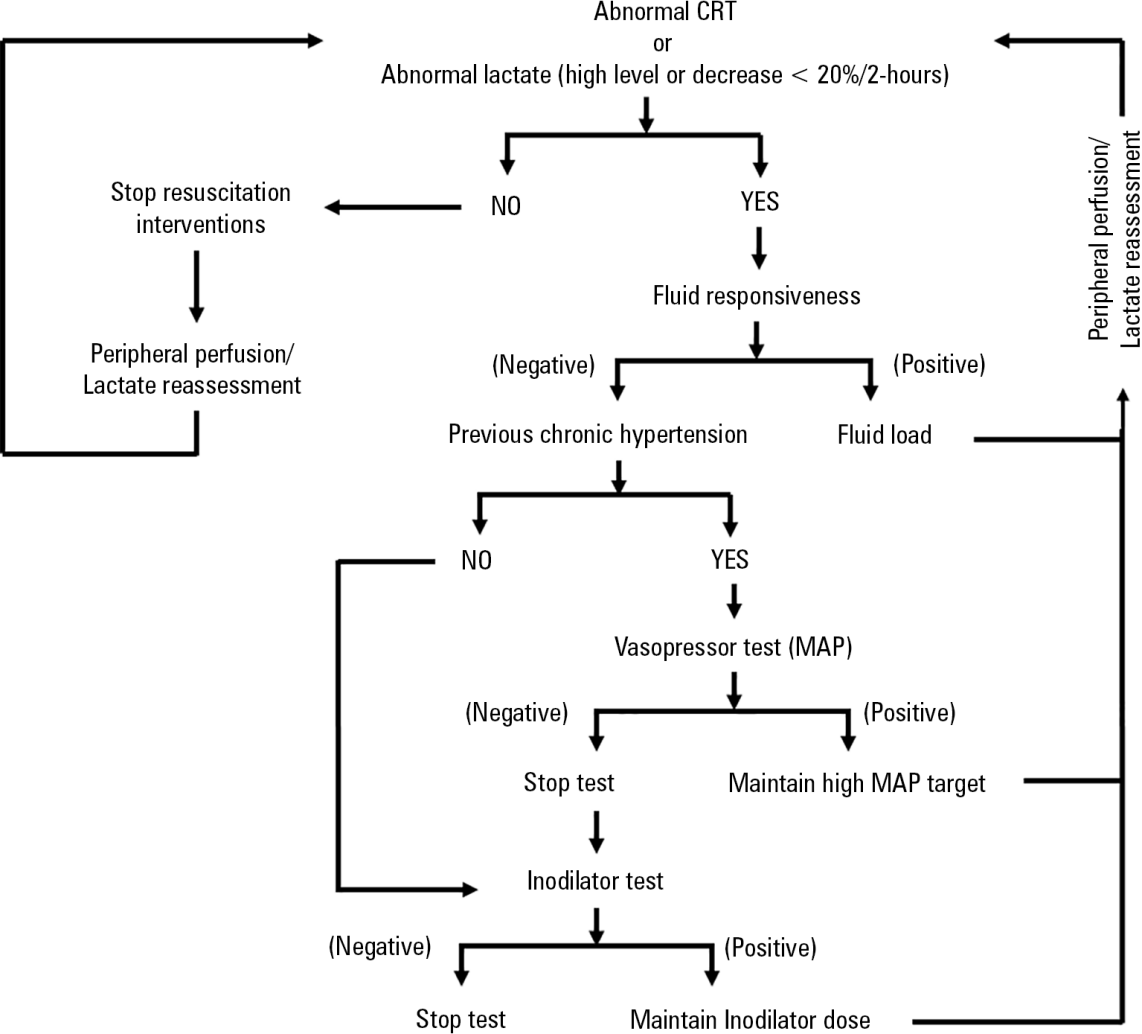
Sequential approach to resuscitation. The process starts with fluid loading according to the status of fluid-responsiveness. If the goal is not obtained, the second step is a vasopressor test and then an inodilator test. CRT - capillary refill time; MAP - mean arterial pressure.

ANDROMEDA-SHOCK is a prospective, multicenter, parallel-group, randomized trial that compares an 8-hour protocol of PPTR *versus* LTR in patients with septic shock.^(10)^ The trial is being conducted in 26 ICUs in Argentina, Chile, Ecuador, Colombia and Uruguay. The trial protocol (version 1.0 from December 2016) was published^(11)^ and is registered with ClinicalTrials.gov (NCT03078712). It was approved by the Ethics Committees of all of the participating institutions. The main study interventions are summarized in figure 1.

### Randomization

Eligible patients will be randomly allocated to the PPTR or LTR Groups. Peripheral perfusion-targeted resuscitation will be aimed to normalize CRT. Lactate-targeted resuscitation will aim to either normalize lactate or decrease it at a rate of more than 20% per 2 hours during the 8 hours of the study period. A randomization sequence with an allocation of 1:1 will be generated by a computer program. Study-group assignment will be achieved by means of randomized permuted blocks of eight (without stratification). Allocation concealment will be maintained by means of central randomization. Investigators at the sites will call a representative at the Study Coordinating Center (SCC), who will be available 24 hours per day and 7 days per week through a dedicated phone number. The group to which the patient is allocated will only be disclosed after the information is recorded by the SCC. Such a measure prevents the investigator and the medical team from predicting which treatment group the patient will be allocated to.

### Study interventions

A sequential approach to resuscitation will be followed in both groups as shown in figure 1. The intervention period covers the first 8 hours following randomization. All other treatments during and after the intervention period will be at the discretion of the treating clinicians according to their local protocols.

In the PPTR Group, CRT will be measured every 30 minutes until normalization. After normalization, it will be measured hourly until the end of the 8 hours protocol. Capillary refill time is measured by applying firm pressure to the ventral surface of the right index distal phalanx with a glass microscope slide. The pressure will be increased until the skin is blanched and then maintained for 10 seconds. The time that it takes to return to the normal skin color will be registered with a chronometer. A CRT > 3 seconds will be considered abnormal.^(12)^

In the LRT Group, lactate will be assessed every two hours during the 8-hour study period.

Fluid responsiveness will be assessed using a structured approach outlined in the protocol, which includes different predictors (passive leg-raising, end-expiratory occlusion test, pulse pressure variation, respiratory variations of the inferior vena cava, and aortic velocity time integral) customized according to the patients' specific conditions (for example, whether the patient is under mechanical ventilation, has irregular cardiac rhythm, acute respiratory distress syndrome (ARDS)/low respiratory-system compliance).

In patients in both groups who are predicted to be fluid-responsive, fluid resuscitation is started (500mL of crystalloids in 30 minutes). Fluid resuscitation is terminated when safety measures are met (i.e., an increase in central venous pressure - CVP ≥ 5mmHg or the patient has become fluid unresponsive) or the endpoint has been reached. In the PPTR Group, the endpoint is the normalization of the CRT. In the LTR Group, the endpoint is either that lactate has normalized or it has decreased > 20% from a previous value.

An open-label vasopressor test will be performed with the aim of increasing mean arterial pressure (MAP) to 80 - 85mmHg using progressively increasing doses of norepinephrine in patients with a previous history of chronic hypertension (as defined by the use of antihypertensive medications before admission and by medical history). The test is performed in both groups when fluid resuscitation does not reach the endpoint (i.e., persistent abnormal CRT or an inadequate decrease in lactate levels) and the patient no longer has signs of fluid-responsiveness or when CVP has increased ≥ 5mmHg. Endpoints will be reassessed after reaching the MAP goal. In patients on the PPTR protocol, endpoints will be reassessed one hour after reaching the MAP goal; patients on the LTR protocol will be reassessed two hours after reaching the MAP goal. If the vasopressor test is successful (i.e., CRT improves, and lactate goals are achieved in PPTR and LTR respectively), norepinephrine will be titrated to maintain this MAP throughout the study period. If the goals are not achieved, if the norepinephrine dose is higher than 0.8mcg/kg/min, or if adverse effects occur (e.g., heart rate > 140bpm, arrhythmias or evident cardiac ischemia), the norepinephrine dose will be reduced to the level that it was before the vasopressor test, and the protocol will move to the next step.

An open-label test of dobutamine (fixed 5mcg/kg/min) or milrinone (fixed 0.25mcg/kg/min), at the discretion of the attending physician, will be started in nonhypertensive patients with persistent abnormal CRT or nonachieved lactate goals in patients with fluid-unresponsiveness or when fluid resuscitation safety measures are met. If the vasopressor test is unsuccessful in previously hypertensive patients, the same open label dobutamine/milrinone test will be performed. The end points will be reassessed, similar to the vasopressor test. If the endpoints are not met, dobutamine/milrinone will be discontinued and no further action will be taken during the study period, except for rechecking fluid responsiveness every hour and restarting fluid challenges when appropriate. When dobutamine or milrinone is effective, the infusion will be maintained throughout the study period. As a safety measure, inodilators will be stopped if the heart rate increases > 15% or if arrhythmias, ischemia or hypotension develop.

The protocol can be stopped at any moment for safety considerations if the attending intensivist observes that the patient has developed unexpected and severe complications or devolves into refractory shock, conditions that under his judgment require liberalization of management.

### Sample size

Mortality in patients who have circulatory dysfunction and increased lactate levels has been shown to exceed 40%.^(12)^ In addition, several studies have shown that abnormal peripheral perfusion is associated with a mortality exceeding 40%, whereas a normal CRT in the early phase of septic shock has been associated with a less than 10% mortality.^(13,14)^ We anticipate a 28-day mortality rate of 45% in the LTR group of our trial.

A total sample size of 420 patients (210 per group) is expected to provide approximately 90% power to detect a reduction in 28-day mortality from 45% to 30% when analyzing the data using the intention-to-treat (ITT) principle, with a two-sided alpha level of 5%. We consider a 15% reduction (33% relative risk reduction) in mortality to have important clinical value, as was observed in earlier resuscitation studies.^(14)^ In addition, this effect size is plausible because limiting fluid administration has been shown to decrease organ failure, the main determinant of death in septic patients.^(8)^

Nevertheless, we used an adaptive approach,^(15)^ which would allow for a sample size re-estimation at a preplanned interim analysis when 75% of the sample has been recruited. The sample size re-estimation was supposed to be conducted by the independent Data and Safety Monitoring Committee (DSMC) only if the size effect observed in the interim analysis was between 10% and 15% absolute reduction in mortality (promising zone) favoring the PPTR over the LTR group.^(15)^ The favorable zone was defined as an absolute difference > 15% (conditional power > 90%) and the unfavorable zone as an absolute difference < 10% (conditional power < 61%) in the interim analysis.

We calculated operational characteristics of this strategy conducting simulations with 200 studies. Without adaptation, the conditional power for the promising zone was between 61% and 90%. In case the study interim analysis fell in the promising zone, adapting the sample size up to 840 patients would increase the conditional power. Considering a true effect size of 15%, the probability of "landing" in the promising zone was 22%, and the mean conditional power would increase to > 90%. Considering a true effect size of 10%, the probability of "falling" in the promising zone was 40%, and the mean conditional power would increase to > 80%.

This interim analysis was performed on February 2nd, 2018, and the DSMC recommended to continue the trial with no modifications.

### Framework

The design of the study is aimed at demonstrating the superiority of PPTR over LTR in terms of 28-day mortality and other secondary and tertiary outcomes.

### Statistical interim analyses

Interim analyses were conducted after the inclusion of the first 100 patients and at 75% of the sample size (300 patients). Only the independent DSMC had access to the results of those analyses. The DSMC is comprised of 5 experienced intensivists and trialists and 1 senior statistician. The DSMC established no *a priori* statistical stopping guidance according to efficacy, safety or futility. The DSMC recommended that the trial should continue without alterations after those analyses.

### Timing of final analysis

All outcomes will be analyzed simultaneously after we have completed the 90-day follow-up of all patients, and the database has been locked.

### Timing of outcome assessments

We will assess outcomes at 8, 24, 48, and 72 hours, at hospital discharge, and at 28 and 90 days.

### Statistical principles

#### Confidence intervals and p values

We will present 95% confidence intervals (95%CI) for effect estimates on all primary and secondary outcomes. All hypothesis tests will be two-sided with an α of 5%. We will not adjust p-values or confidence intervals for analyses of primary or secondary outcomes. Therefore, all results for secondary outcomes should be interpreted as exploratory.

### Adherence and protocol deviations

We will report the numbers and percentages of nonadherence to the randomly allocated treatments.

Protocol deviations will be assessed and registered by the local coordinators at each center. Major deviations are defined as wrong inclusion (misjudgment of inclusion or exclusion criteria) or inadequate resuscitation procedures during the study period.

### Analysis populations

All analyses will be conducted according to the intention-to-treat principle. Thus, patients will be analyzed in the groups to which they were randomly assigned.

### Trial population

#### Screening data

An active daily screening for potentially eligible patients will be performed at all of the participating ICUs. Screened patients include all patients admitted to the participating ICUs with septic shock criteria or those who develop these criteria during their ICU stay.^(10)^ Patients will be either included or excluded for the study, and the reasons for the latter will be registered and communicated to the SCC on a weekly basis.

#### Eligibility

Consecutive adult patients (≥ 18 years old) with septic shock admitted to the intensive care unit will be considered eligible. Septic shock is defined as suspected or confirmed infection, plus hyperlactatemia (≥ 2.0mmol/L) and the required use of vasopressors to treat refractory hypotension.^(10)^ This latter is characterized as a systolic blood pressure (SBP) < 90mmHg or a MAP < 65mmHg after an intravenous fluid load of at least 20mL/kg, administered over the course of 60 minutes.

Patients will be excluded in the case of pregnancy; anticipated surgery or dialysis procedure during the first 8 hours after a septic shock diagnosis; do-not-resuscitate status; active bleeding; acute hematological malignancy; concomitant severe ARDS; and a duration of more than 4 hours after the onset of septic shock criteria.

### Recruitment

**Figure 2:**
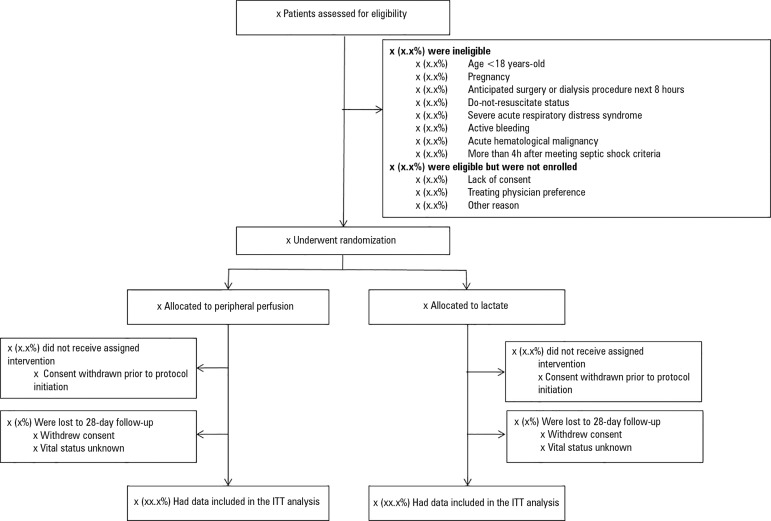
Flow of patients in the ANDROMEDA-SHOCK trial. ITT - intention-to-treat.

Information that will be included in the CONSORT flow diagram is shown in figure 2.

### Withdrawal/follow-up

We will tabulate the number of patients whose consent for trial participation is withdrawn either by the patient or his or her legal representative. When consent is withdrawn for trial participation, we will nevertheless attempt to obtain consent for collecting and analyzing follow-up data. These cases should also be reported.

### Baseline patient characteristics

The baseline characteristics to be registered during the trial will be presented as in mock table 1.

**Table 1:** Baseline characteristics of the patients

Characteristic	Peripheral perfusion-targeted resuscitation (n = xxx)	Lactate-targeted resuscitation (n = xxx)
Age (years)	xx.x (xx.x)	xx.x (xx.x)
Women	xxx (xx.x)	xxx (xx.x)
Charlson comorbidity score	xx (xx to xx)	xx (xx to xx)
APACHE-II	xx (xx to xx)	xx (xx to xx)
SOFA	xx (xx to xx)	xx (xx to xx)
Septic shock source		
Pneumonia	xxx (xx.x)	xxx (xx.x)
Urinary tract infection	xxx (xx.x)	xxx (xx.x)
Intra-abdominal infection	xxx (xx.x)	xxx (xx.x)
Skin or soft-tissue infection	xxx (xx.x)	xxx (xx.x)
Other source	xxx (xx.x)	xxx (xx.x)
Infection of unknown source	xxx (xx.x)	xxx (xx.x)
Hemodynamic and perfusion-related variables		
Heart rate (bpm)	xx.x (xx.x)	xx.x (xx.x)
Mean arterial pressure (mmHg)	xx.x (xx.x)	xx.x (xx.x)
Norepinephrine dose (mcg/kg/min)	x.xx (x.xx)	x.xx (x.xx)
Central venous pressure (mmHg)	xx.x (xx.x)	xx.x (xx.x)
Serum lactate (mmol/L)	x.xx (x.xx)	x.xx (x.xx)
Central venous oxygen saturation	xx.x (xx.x)	xx.x (xx.x)
Venous-arterial PaCO_2_ gradient (mmHg)	xx.x (xx.x)	xx.x (xx.x)
Capillary refilling time (sec)	x (x to x)	x (x to x)
Mottling score	x (x to x)	x (x to x)
Initial management data		
Time from matching entry criteria to randomization (min)	xx (xx)	xx (xx)
Intravenous fluid loading before randomization (mL)	xxxx (xxxx)	xxxx (xxxx)
Time from diagnosis of septic shock to first antibiotics (min)	xxx (xxx)	xxx (xxx)

SOFA - Sepsis Organ Failure Assessment; APACHE - Acute Physiology and Chronic Health Evaluation; PaCO_2_ - partial pressures of carbon dioxide. Values expressed as number (%), mean (standard deviation), or median (interquartile range).

### Analysis

#### Outcome definitions

Our primary outcome is all-cause mortality within 28 days.

Our secondary outcomes are:

- All-cause mortality within 90 days.
- Mechanical ventilation-free days during the first 28 days after randomization. A day free of mechanical ventilation is defined as no need of invasive mechanical ventilation at any time during a given day.
- Renal replacement therapy-free days during the first 28 days after randomization.
- Vasopressor-free days during the first 28 days after randomization.
- Organ dysfunction, as assessed by the Sepsis Organ Failure Assessment (SOFA) score at 72 hours after randomization.^(16)^
- ICU and hospital length of stay, truncated at 90 days.

Our tertiary exploratory outcomes are:

- Amount of resuscitation fluids administered in the first 8 and 24 hours after randomization.
- Total fluid balance in the first 8, 24, 48 and 72 hours.
- Occurrence of intra-abdominal hypertension during the first 72 hours after randomization (%).
- Use of renal replacement therapy (%) within 28 days.
- In-hospital mortality, truncated at 90 days.

The protocol did not call for systematic measurement of intra-abdominal pressure. Therefore, intra-abdominal pressure was measured according to physicians' discretion when they suspected intra-abdominal hypertension.

### Analysis methods

Continuous distribution will be assessed by visual inspection of histograms and D'Agostino-Pearson's normality tests. Variables will be expressed as counts and percentages, the mean and standard deviation (SD), or the median and interquartile range (IQR). Whenever appropriate, this is shown in mock tables 1 to 3, which we intend to include in the main results paper.

**Table 3:** Outcomes of patients treated with peripheral perfusion-targeted resuscitation versus lactate-targeted resuscitation

Outcome	Peripheral perfusion-targeted resuscitation (n=xxx)	Lactate-targeted resuscitation (n=xxx)	Type of effect estimate	Effect estimate (95%CI)	p value
Primary outcome					
Death within 28 days	xx (xx.x)	xx (xx.x)	Hazard ratio	x.xx (x.xx to x.xx)	x.xx
Secondary outcomes					
Death within 90 days	xx (xx.x)	xx (xx.x)	Hazard ratio	x.xx (x.xx to x.xx)	x.xx
Mechanical ventilation-free days within 28 days	xx.x	xx.x	Mean difference	x.x (x.x)	x.xx
Renal replacement therapy-free days within 28 days	xx.x	xx.x	Mean difference	x.x (x.x)	x.xx
Vasopressor-free days within 28 days	xx.x	xx.x	Mean difference	x.x (x.x)	x.xx
SOFA					
SOFA at 8 hours	x.x	x.x	Mean difference	x.x (x.x)	x.xx
SOFA at 24 hours	x.x	x.x	Mean difference	x.x (x.x)	x.xx
SOFA at 48 hours	x.x	x.x	Mean difference	x.x (x.x)	x.xx
SOFA at 72 hours	x.x	x.x	Mean difference	x.x (x.x)	x.xx
ICU length of stay (days)	x.x	x.x	Mean difference	x.x (x.x)	x.xx
Hospital length of stay (days)	x.x	x.x	Mean difference	x.x (x.x)	x.xx
Tertiary outcomes					
Amount resuscitation fluids (mL)					
At 8 hours	xxxx (xxxx)	xxxx (xxxx)	Mean difference	xxx (xxx to xxx)	x.xx
At 24 hours	xxxx (xxxx)	xxxx (xxxx)	Mean difference	xxx (xxx to xxx)	x.xx
Total fluid balance (mL)					
At 8 hours	xxxx (xxxx)	xxxx (xxxx)	Mean difference	xxx (xxx to xxx)	x.xx
At 24 hours	xxxx (xxxx)	xxxx (xxxx)	Mean difference	xxx (xxx to xxx)	x.xx
At 72 hours	xxxx (xxxx)	xxxx (xxxx)	Mean difference	xxx (xxx to xxx)	x.xx
Intra-abdominal hypertension	xx (x.x)	xx (x.x)	Risk difference	x.x (x.x to x.x)	x.xx
Use of renal replacement therapy	xx (x.x)	xx (x.x)	Risk difference	x.x (x.x to x.x)	x.xx
In-hospital mortality	xxx (xx.x)	xxx (xx.x)	Risk difference	x.x (x.x to x.x)	x.xx

95%CI - 95% confidence interval; ICU - intensive care unit; SOFA - Sepsis Organ Failure Assessment. Values expressed as number (%) or mean (standard deviation).

The evolution of hemodynamic and perfusion variables in both groups during the study will be presented in mock table 2. We will carry out linear mixed models for continuous variables to account for the repeated measurements on the same patient. Binary variables will be tested using logistic mixed regression models and for continuous variables with nonsymmetrical distributions, such as Lactate and Mottling scores, we will use the distribution that best fits the data.

**Table 2:** Evolution of hemodynamic and perfusion variables from baseline to 72 hours in the peripheral perfusion-targeted resuscitation and lactate-targeted resuscitation groups

Variable	Group	Basal	2 hours	4 hours	8 hours	24 hours	48 hours	72 hours
Number of patients	PPTR	xxx	xxx	xxx	xxx	xxx	xxx	xxx
	LTR	xxx	xxx	xxx	xxx	xxx	xxx	xxx
Heart rate (bpm), mean	PPTR	xxx	xxx	xxx	xxx	xxx	xxx	xxx
	LTR	xxx	xxx	xxx	xxx	xxx	xxx	xxx
	p-value	-	x.xx	x.xx	x.xx	x.xx	x.xx	x.xx
Systolic blood pressure (mmHg), mean	PPTR	xxx	xxx	xxx	xxx	xxx	xxx	xxx
	LTR	xxx	xxx	xxx	xxx	xxx	xxx	xxx
	p-value	-	x.xx	x.xx	x.xx	x.xx	x.xx	x.xx
Diastolic blood pressure (mmHg), mean	PPTR	xx	xx	xx	xx	xx	xx	xx
	LTR	xx	xx	xx	xx	xx	xx	xx
	p-value	-	x.xx	x.xx	x.xx	x.xx	x.xx	x.xx
Mean arterial pressure (mmHg), mean	PPTR	xx	xx	xx	xx	xx	xx	xx
	LTR	xx	xx	xx	xx	xx	xx	xx
	p-value	-	x.xx	x.xx	x.xx	x.xx	x.xx	x.xx
Norepinephrine dose (mcg/kg/min), mean	PPTR	x.xx	x.xx	x.xx	x.xx	x.xx	x.xx	x.xx
	LTR	x.xx	x.xx	x.xx	x.xx	x.xx	x.xx	x.xx
	p-value	-	x.xx	x.xx	x.xx	x.xx	x.xx	x.xx
Norepinephrine use, n (%)	PPTR	xx (xx.x)	xx (xx.x)	xx (xx.x)	xx (xx.x)	xx (xx.x)	xx (xx.x)	xx (xx.x)
	LTR	xx (xx.x)	xx (xx.x)	xx (xx.x)	xx (xx.x)	xx (xx.x)	xx (xx.x)	xx (xx.x)
	p-value	-	x.xx	x.xx	x.xx	x.xx	x.xx	x.xx
Diuresis (total mL in previous period), mean	PPTR	-	xxx	xxx	xxx	xxx	xxx	xxx
	LTR	-	xxx	xxx	xxx	xxx	xxx	xxx
	p-value	-	x.xx	x.xx	x.xx	x.xx	x.xx	x.xx
Lactate (mmol/L), mean	PPTR	x.xx	x.xx	x.xx	x.xx	x.xx	x.xx	x.xx
	LTR	x.xx	x.xx	x.xx	x.xx	x.xx	x.xx	x.xx
	p-value	-	x.xx	x.xx	x.xx	x.xx	x.xx	x.xx
Capillary refill time (sec), median	PPTR	x	x	x	x	x	x	x
	LTR	x	x	x	x	x	x	x
	p-value	-	x.xx	x.xx	x.xx	x.xx	x.xx	x.xx
Central venous oxygen saturation, mean, %	PPTR	xx	-	-	xx	xx	xx	xx
	LTR	xx	-	-	xx	xx	xx	xx
	p-value	-	-	-	x.xx	x.xx	x.xx	x.xx
Delta PaCO_2_ (mmHg), mean	PPTR	xx.x	-	-	xx.x	xx.x	xx.x	xx.x
	LTR	xx.x	-	-	xx.x	xx.x	xx.x	xx.x
	p-value	-	-	-	x.xx	x.xx	x.xx	x.xx
Mottling score, median	PPTR	x	-	-	x	x	x	x
	LTR	x	-	-	x	x	x	x
	p-value	-	-	-	x.xx	x.xx	x.xx	x.xx

PPTR - peripheral perfusion-targeted resuscitation; LTR - lactate-targeted resuscitation; delta PaCO_2_ - central venous-arterial PaCO_2_ gradient.

We will assess the effect of PPTR *versus* LTR on the primary outcome using Cox proportional hazards models, with adjustment for 5 prespecified baseline covariates: *Acute Physiology and Chronic Health Evaluation* (APACHE II) score, SOFA score, lactate level, CRT and source of infection, as fixed (individual-level) effects. The results will be reported as hazard ratios with 95%CI and p-values. We should also present Kaplan-Meier curves.

Effects on secondary and tertiary outcomes will be presented as a hazard ratio for 90-day all-cause mortality and renal replacement therapy within 28 days or the risk difference for all other binary outcomes, along with 95%CI and p-values (calculated with Fisher's exact tests), as shown in mock table 3. The effect on 90-day all-cause mortality and the need for renal replacement therapy within 28 days will be assessed with a Cox-proportional hazard model without adjustment for baseline covariates.

We will estimate the effect on mechanical ventilation-free days, renal replacement therapy-free days and vasopressor-free days for 28 days with generalized linear models using the distribution that best fits the data (possibly truncated Poisson distribution). Effects on organ dysfunction at 72 hours (as measured by SOFA) will be calculated with generalized linear models with the distribution that best fits the data, with adjustment for the baseline SOFA. The effect on other continuous outcomes, such as ICU or hospital length of stay, amount of resuscitation fluids administered, and fluid balance will also be calculated with generalized linear models with the distribution that best fits the data (normal, gamma, inverse Gaussian, or other), without adjustment for covariates.

### Subgroup analyses

We will use a Cox proportional hazards model adjusted for baseline covariates (the same as for the main analysis) to assess interactions between treatment effects and the following prespecified subgroups: a) patients with lactate > 4.0mmol/L versus equal to or less than 4mmol/L; b) patients without a confirmed source of infection (as this could erroneously include other critically ill patients) versus those with a confirmed source of infection; c) patients with APACHE II scores less than *versus* equal to or greater than 25; d) patients with a SOFA score less than *versus* equal to or greater than 10; e) patients with a more than 10% difference in lactate levels between the very first one measured and the baseline when starting the study.

### Sensitivity analysis

We will assess the effect of PPTR compared to LTR on 28-day mortality using a frailty Cox model with site as the random effect, and we will adjust for the same baseline covariates as in the main analysis (APACHE II score, SOFA score, lactate level, CRT and source of infection).

### Harms

Our primary, secondary and tertiary outcomes are intended to reflect potential harms resulting from using the PPTR *versus* LTR approach for managing septic shock.

### Missing data

The primary outcome (28-day mortality) will be treated as a time-to-event outcome and reported in Cox proportional hazard models; patients with no follow-up information will be recorded at the last point of contact. We will use multiple imputation methods to assess the treatment effect on the primary outcome if there are cases with no follow-up information at all. As a sensitivity analysis, we will also assess the effect on the primary outcome using complete case data.

### Statistical software

Analyses will be performed using the R (R Core Team, 2017, Vienna, Austria) software.

## CONCLUSION

According to the best trial practice, we report our statistical analysis plan and data management plan prior to locking the database and starting analyses. We anticipate that this practice will prevent analysis bias and enhance the utility of the reported results.

### Authors' contributions

G Hernández, AB Cavalcanti, and J Bakker are guarantors of the entire manuscript; J Bakker, JL Teboul, G Hernández, G Ospina-Tascón, A Dubin, G Friedman, M Cecconi, FJ Hurtado, AB Cavalcanti, R Castro, L Alegría, and LP Damiani designed the study. All of the authors will help in the data interpretation and the final manuscript draft. All authors read and approved this final manuscript.

### ANDROMEDA-SHOCK investigators include

**Writing and Steering Committee:** Glenn Hernandez (chair), Gustavo Ospina-Tascón, Alexandre Biasi Cavalcanti, Arnaldo Dubin, Francisco Javier Hurtado, Gilberto Friedman, Ricardo Castro, Leyla Alegría, Jean-Louis Teboul, Maurizio Cecconi, Lucas Petri Damiani, and Jan Bakker (cochair).
